# Prosthodontic treatment of patients with ectodermal dysplasia

**DOI:** 10.1186/1746-160X-8-S1-I5

**Published:** 2012-05-25

**Authors:** Heiner Weber

**Affiliations:** 1 Department of Prosthodontics, University Hospital Tübingen, Germany

## 

The definition of “health” as provided by the World Health Organization (WHO) points out that this status is not only given by the absence of disease and infirmity – but that social well-being is an inherent parameter for a healthy subject. Within medicine and dentistry, individuals with ectodermal dysplasia represent a very special group of patients. Diagnostics and treatment planning have to start early in childhood. Furthermore, this disease requires the early cooperation of three disciplines of our profession: prosthodontics to define the final outcome at a later, adolescent age, and both orthodontics and maxillofacial surgery to accompany and treat the patients in an ongoing/stand-by way. Treatment decisions of the dental team depend on the patients’ needs, wishes, their willingness to undergo minor or major treatment with different impact, and also on the economic possibilities. Therapeutic options normally range from lining up existing teeth according to a final treatment plan to preliminary dentures and – at the end – to bone grafting and dental implants, often followed by extensive restorative/prosthetic therapies. Different prosthetic devices may be indicated and the patients need to be encouraged for an appropriate treatment.

